# Consistency, variability, and predictability of on-farm nutrient responses in four grain legumes across East and West Africa

**DOI:** 10.1016/j.fcr.2023.108975

**Published:** 2023-08-01

**Authors:** Joost van Heerwaarden, Esther Ronner, Frederick Baijukya, Samuel Adjei-Nsiah, Peter Ebanyat, Nkeki Kamai, Endalkachew Wolde-meskel, Bernard Vanlauwe, Ken E. Giller

**Affiliations:** a Plant Production Systems, Wageningen University, P.O.Box 430, 6700 AK Wageningen, the Netherlands; b International Institute of Tropical Agriculture (IITA), P.O. Box 3444, Dar es Salaam, Tanzania; c International Institute of Tropical Agriculture, CSIR Campus, Accra, Ghana; d Department of Agricultural Production, Makerere University, P.O. Box 7062, Kampala, Uganda; e Department of Crop Production, Faculty of Agriculture, University of Maiduguri, Maiduguri, Nigeria; f World Agroforestry (ICRAF), C/o International Livestock Research Institute, Box 5689, Addis Ababa, Ethiopia; g IITA, Natural Resource Management Research Area, Nairobi, Kenya

**Keywords:** Soil fertility, Sub Saharan Africa, Nutrient response variability, Legumes

## Abstract

Grain legumes are key components of sustainable production systems in sub-Saharan Africa, but wide-spread nutrient deficiencies severely restrict yields. Whereas legumes can meet a large part of their nitrogen (N) requirement through symbiosis with N_2_-fixing bacteria, elements such as phosphorus (P), potassium (K) and secondary and micronutrients may still be limiting and require supplementation. Responses to P are generally strong but variable, while evidence for other nutrients tends to show weak or highly localised effects. Here we present the results of a joint statistical analysis of a series of on-farm nutrient addition trials, implemented across four legumes in four countries over two years. Linear mixed models were used to quantify both mean nutrient responses and their variability, followed by a random forest analysis to determine the extent to which such variability can be explained or predicted by geographic, environmental or farm survey data. Legume response to P was indeed variable, but consistently positive and we predicted application to be profitable for 67% of farms in any given year, based on prevailing input costs and grain prices. Other nutrients did not show significant mean effects, but considerable response variation was found. This response heterogeneity was mostly associated with local or temporary factors and could not be explained or predicted by spatial, biophysical or management factors. An exception was K response, which displayed appreciable spatial variation that could be partly accounted for by spatial and environmental covariables. While of apparent relevance for targeted recommendations, the minor amplitude of expected response, the large proportion of unexplained variation and the unreliability of the predicted spatial patterns suggests that such data-driven targeting is unlikely to be effective with current data.

## Highlights

• A large number of on-farm fertilizer trials show consistent but variable responses to phosphorus.
• No consistently positive effect was found for potassium or secondary and micronutrients.
• Potassium response shows spatial variation, but its small magnitude and lack of predictability limit the scope for targeted recommendations.

## 1 Introduction

Grain legumes are important for sustainable intensification of agriculture, particularly on small farms in sub-Saharan Africa (SSA) (Droppelmann et al., 2017, Snapp et al., 2019, Vanlauwe et al., 2019b). They fit into a diversity of farming systems as monocrop, in rotation or as relay- or intercrop (Snapp et al., 2019, Thierfelder et al., 2012, Vanlauwe et al., 2019b) and provide benefits in terms of soil fertility, soil cover, pest and disease control and as source of food, feed and income (Franke et al., 2018, Muoni et al., 2019, Snapp et al., 2019). A unique advantage is their ability to obtain between 10% and 90% of nitrogen demand through symbiosis with nitrogen fixing rhizobia (Franke et al., 2018), reducing the need for nitrogen (N) fertiliser. Unfortunately, productivity of legumes in SSA remains constrained by low soil fertility (Kermah et al., 2018, Ojiem et al., 2007, Wortmann et al., 2019), on top of other problems like biotic stresses, poor seed quality and drought (Giller, 2001, Waddington et al., 2010). As a result, yields in SSA often remain below 25% of their water limited potential (van Loon et al., 2018).

The application of nutrients such as phosphorus (P), potassium (K), secondary (Ca, Mg, S) and micronutrients (B, Zn, Mo) has been proposed as a solution to soil fertility constraints in SSA (Kihara et al., 2017, Vanlauwe et al., 2019a, Wortmann et al., 2019), as has the use of rhizobium inoculants (Vanlauwe et al., 2019a). For inoculants, yield gains of about 100 kg ha^−1^ and cost-effectiveness for over 90% of farmers was recently found for soybean across SSA (van Heerwaarden et al., 2018). Evidence for other legumes is less clear-cut but positive responses have been reported in common bean (Amijee and Giller, 1998, Ndakidemi et al., 2006), chickpea (Wondwosen et al., 2016) and cowpea (Boddey et al., 2017, Kyei-Boahen et al., 2017). Since inoculants tend to be cheap they may be considered a low-risk recommendation, unlike mineral fertilisers, whose high purchase costs require distinct yield benefits to be offset.

In that regard, it is worth noting that despite well-documented legume responses to phosphorus (Mucheru-Muna et al., 2010, Vesterager et al., 2008, Wolde-meskel et al., 2018), particularly in soybean (Kamara et al., 2007, Kolawole, 2012, Zingore et al., 2008), most reports only cover individual regions, with considerable variation among studies and locations (Ronner et al., 2016, Ulzen et al., 2018). Such inconsistent responses have implications for the cost-effectiveness and risk of nutrient application and deserve further investigation, ideally across different regions and years. Evidence for benefits of other nutrients such as potassium are scarce by comparison (Kihara et al., 2017), and the few published studies tend to include only a small number of locations, predominantly in pot trials or on-station (Bado et al., 2006, Keino et al., 2015). A recent exception describing on-farm and on-station responses to combined application of Mg, S, Zn and B across SSA (Wortmann et al., 2019) found that among crop types, legumes had the smallest relative increases in yield. Similar small and non-consistent effects were found across different countries for S and micronutrients in soybean and cowpea (Kaizzi et al., 2012, Kihara et al., 2017) and for K in common bean (Kaizzi et al., 2018), suggesting limited potential for a profitable return to the investment. Hence, the magnitude and consistency of agronomic and/or economic benefits of the addition of P, K and other nutrients in smallholder legume production remains an important topic for research.

Here, we analyse a recently compiled dataset of on-farm nutrient addition trials that were set up to evaluate the additive effects of phosphorus (P), potassium (K), and a variety of secondary and micronutrients (abbreviated as SMN here) in four legume crops: soybean (*Glycine max*), groundnut (*Arachis hypogaea*), cowpea (*Vigna unguiculata*), and climbing bean (*Phaseolus vulgaris*), across four countries in East and West Africa (Tanzania, Uganda, Nigeria and Ghana) with the purpose of assessing the general response to these nutrients and to describe the magnitude and patterns of variation. We thereby distinguish different types of variation of contrasting agronomic relevance. The first distinction is between systematic and non-systematic variation. Non-systematic variation is the component of observed variability due to random effects of sampling and experimental error at the experimental plot level which is highly localised, non-repeatable and hence of limited relevance (van Heerwaarden et al., 2018, Vanlauwe et al., 2019a). Systematic variation, on the other hand, reflects differences in growing conditions at the field level and above. This type of response variation is experienced by farmers across locations and time points and is therefore of direct agronomic importance. A second distinction is made between explainable and predictable systematic variation. Explainable variation is associated with known climatic, edaphic, weather, biotic stress or crop management conditions and offers opportunities for adapting the amount and composition of inputs to local circumstances. Such adaptation is only possible when responses are also predictable, i.e., calculated before the growing season starts. In practice, much of the explainable variation is likely to be due to unpredictable growing conditions and constitutes production risk that may negatively affect farmer’s willingness to invest in inputs.

In summary, the present study aims to establish the main effects of nutrient application in grain legumes and to dissect the different types of systematic response variation that are relevant for understanding production risks and potential for tailored application (i.e. predictable variation). We address the following research questions: First, what are the average summative effects of P, K and secondary and micronutrient application in the studied areas? Second, what is the magnitude of systematic response variation and how is this variation distributed in space and time? Third, is the economic risk associated with systematic variation in nutrient response small enough to permit general nutrient recommendations? Finally, can variation in response to nutrients be predicted from geographical and environmental data, to allow site-specific recommendations? By answering these questions, we evaluate the potential to raise yields and to mitigate production risk using available knowledge, which is of general interest to similar systems elsewhere.

## 2 Methods

### 2.1 Data

**Fig. 1:**
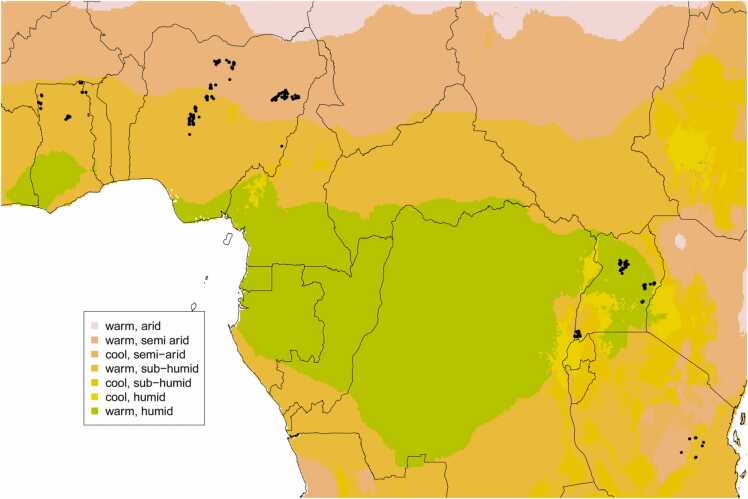
Map showing trial sites used in this study (black dots). Coloured shading marks different agro-ecological zones (Harvest choice, https://doi.org/10.7910/DVN/M7XIUB).

The data was taken from a total of 399 on-farm trials performed in Ghana, Nigeria, Uganda and Tanzania (Fig. 1) from 2015 to 2017 and covering the crops soybean, groundnut, cowpea and climbing bean, yielding a total of 2523 data points. Rainfall in Uganda and Tanzania is bimodal, which in the case of Uganda translated into a wide range of planting dates within a year. Although trials in different countries differed in exact treatment structure, varieties, agronomic practices and nutrient formulations, all were researcher-managed, non-replicated, on-farm experiments which included a zero-input control and at least three plots with cumulative additions of phosphorus, potassium and secondary and micronutrients (of various kinds, Table S1). Phosphorus was applied at an average rate of 15 kg P ha^−1^ across fields (10–30 kg P ha^−1^, 18 kg P ha^−1^ averaged across experiments) as either single super phosphate (SSP) or triple super phosphate (TSP) and potassium was applied at 32 kg K ha^−1^ (17–60 kg K ha^−1^, 27 kg K ha^−1^ averaged across experiments) as muriate of potash (MOP). The structure of the treatments did not allow an assessment of the effects of individual secondary and micronutrients which is why they are treated as single treatment (SMN).

For each on-farm trial, household survey data was available from which agronomically relevant variables were obtained to be used as co-variates. Digital maps with weather, climatic and soil variables were also compiled for this purpose. Weather variables consisted of monthly satellite-based rainfall data (rain) for the relevant growing seasons, climatic variables were so-called bioclimatic variables derived from long-term temperature (temp) and precipitation (prec) data (Fick and Hijmans, 2017). Soil variables (soil) consisted of predicted physical and chemical properties (see Table S2 for full details on all variables).

### 2.2 Statistical methods

Using the combined data across countries, crops and years, a statistical model was implemented that allowed: 1) the estimation and testing of mean effects of the different types of nutrients and 2) the quantification and dissection of random response variation into different components of variation. To this aim, linear mixed models were used. This type of model contains fixed effect terms to represent factors for which the means of the individual levels are of direct interest, as well as random effects to represent the variation around the estimated means. Random effect terms can be specified to account for different sources of variation. Variance components are estimated for each term, quantifying the associated variance, and random effect estimates for each individual factor level. The latter estimates are known as linear unbiased predictors (BLUPs) and their inspection provides insights on which specific factor levels contribute most to the random variation. Specifically, the following model was fitted:

(1) yield∼crop* (p + k + smn)+experiment/(p + k + smn)+ crop:district/(p + k + smn)+ crop:district:year/(p + k + smn)+ field/(p + k + smn)+ om+n + i + g+l+error
  The terms p, k and smn represent phosphorus, potassium, and secondary and micronutrients, respectively, which are the main treatments of interest. Other, non-standard, treatments applied in subsets of experiments were corrected for by including them as random effects with om, n, i, g and l indicating the application of manure, nitrogen, inoculant, gypsum or lime respectively. The “* symbol indicates that both main effects and interactions are considered.
  Random terms in the model are underlined and represent different components of spatial and temporal variation. The “/” symbol is a nesting operator which ensures that random variation for control yield and nutrient responses is modelled individually for each component of random spatial and temporal variation. The error term is the plot-level residual, representing non-systematic variation due to within-field heterogeneity and experimental error. The remaining random terms correspond to different sources of systematic variation in control yield and nutrient response. The following sources of systematic random variation are represented in the model: 1) “experiment”, which is the combination country, year and crop, and represents groups of on-farm trials with an identical set-up and treatment structure, 2) district within crop (district:crop), 3) year within district within crop (district:crop:year), and 4) field, where individual fields are not replicated and are unique to a specific year.
  This model specification allows year-to-year variation to be separated from spatial differences between districts, some of which were only sampled in a single year. Fields were not replicated, so the field random term would normally represent the residual error in the statistical model. Estimation of residual within-field variation therefore required some simplifying model assumptions; by assuming a single effect across fields for non-standard treatments (om, n, i, g and l), plots with such treatments could be used as *de facto* within-field replicates to obtain an approximate estimate of residual within-field variation.
  To gain insight into the contribution of individual random factor levels (e.g. specific districts, fields) to random variation in yield and nutrient responses, principal component analysis (PCA) was applied to the BLUPs (i.e. random effect estimates) corresponding to the different components of random variation, and the first two components were visualised with biplots. Where relevant, data subsets for specific countries or experiments were further analysed with simplified mixed models with field as random effect and year or district as fixed effects, to look more in depth into specific interactions associated with patterns observed in the biplots.
  Among the random terms in the mixed model, only districts had replicates in time which means that, in contrast to the other terms, this component (district:crop) represents variation that can be predicted and managed in theory. Variation associated with the remaining components (experiment, district:crop:year, field) are unpredictable without additional knowledge and may therefore be considered representative of the production risk that farmers face. One step to reducing such production risk is to use a statistical model that predicts random nutrient response variation as a function of underlying climatic, soil or agronomic conditions.
  We explored such an approach here in an attempt to explain and predict a maximum amount of the total field-level variation in control yield and nutrient responses. As a first step we fitted the following, simplified mixed effects model:
(2) yield∼ p + k + smn + (p + k + smn|crop:country) + (p + k + smn|field)+om+ n + i + g+l+error
  to allocate all random response variation below the crop:country level to a fieldlevel random effects term. The operator “|” indicates that for each crop:country and field, specific random effects and interactions were estimated for the three types of nutrients. The BLUPs extracted from this model, representing the field-level deviations for control yield and P, K and SMN response, were saved and merged with the corresponding household survey, weather, climatic and soil data described above (Table S2).

All variables were categorised as either explanatory or predictive. Predictive variables are defined as those whose values are known before the season in which yield was measured, such as climatic and soil properties and farm and field characteristics such as farm size, land ownership and perceived soil fertility. Explanatory variables alsoencompass information which is only known after planting and harvest, such as rainfall, agronomic management and pest and disease occurrence.

Subsequently, a random forest (Breiman, 2001) machine learning algorithm was implemented to model the field-level random variation in yield and response as a function of these predictive and explanatory variables, either together or as subsets. Goodness of fit of the random forest models was summarised by out of bag (OOB) R squared, which should avoid overfitting. It is becoming common to use machine learning models such as these to map the spatial distribution of predicted yield and response (Bonilla-Cedrez et al., 2021, Cao et al., 2021, Kinane et al., 2021) and we used the same approach here. While attractive as a way of identifying potential recommendation domains, such spatial predictions may be subject to large prediction uncertainty and spatial biases, specifically when data is not sampled randomly, as is often the case with on-farm data.

We tackle these limitations in two ways. First, we used a statistical approach to delineate the spatial area for which the on-farm locations are considered to be representative (Nziguheba et al., 2021). A random forest model was trained to distinguish between trial locations and random map coordinates, based on the set of predictive geospatial variables used for the yield and response predictions. Using this model, the probability of representing a potential trial location was calculated for each pixel on the map, with pixels having higher probabilities being most like the sampled on-farm locations. A representative area was then defined as the set of pixels for which the probability was larger than the lowest 1% of site probabilities found among the original on-farm trial locations, which corresponded to a probability of 0.53 in this case. Second, since explanatory variables tend to be spatially correlated, predicted patterns may inadvertently reflect unobserved heterogeneity in local conditions and trial implementation rather than true environmental contrasts. We therefore compare spatial predictions based on a full set of environmental variables to those based on coordinates only. A large part of the variation explained by geographic coordinates alone suggests that the predictive power of environmental variables may be partially related to the unobserved heterogeneity instead of having actual predictive value. In such cases, evaluating the consistency between the two spatial models can provide a further indication of the reliability of the site-specific predictions.

### 2.3 Analysis of economic benefit and risk

The BLUPs representing the field-level deviations in P response derived from the second mixed model were combined with input and grain price information to perform an analysis of economic benefits and risks. Costs for the different types of phosphorus fertiliser (SSP, DAP or TSP) were collected from www.Africafertilizer.org for Nigeria, Tanzania and Uganda, based on the availability of data between 1-1–2015 and 31–12–2017 (the study period). For Ghana, costs for TSP were derived from IFDC (2019). As much as possible, regional retail prices for the different types of fertilisers were considered and expected to represent the spatial variation in fertiliser prices as described by Bonilla Cedrez et al. (2020). For Ghana, only a national average retail price for TSP was available. Costs for transport from the point of sale to the homestead were assumed to be 0.05 USD per kg of fertilizer, the average transport cost found by Bonilla Cedrez et al. (2020).

Legume grain prices were derived from national market information systems (www.tridge.com for Nigeria, www.esoko.com for Ghana, www.agmis.infotradeuganda.com for Uganda) and Temu et al. (2014) for Tanzania. These prices represent wholesale market prices found on urban markets. Regional information was often missing, and therefore we considered an average annual price per country, per legume. The wholesale market prices were converted to farm gate prices for a better comparison with fertiliser costs. On average, farm gate prices were found to be 40–70% of wholesale market prices for different legumes assessed in studies in Rwanda, DR Congo, Malawi and Tanzania (Birachi, 2012, Langyintuo et al., 2003, Rusike et al., 2013). An overall average of 60% was applied across countries to convert market prices to farm gate prices.

Fertiliser costs and legume grain prices were converted from national currency to inflation-adjusted purchasing power parity in US dollars (Bonilla Cedrez et al., 2020). National currencies from different years were first divided by the consumer price index (CPI) to adjust for inflation, with 2017 as a reference year. Values were then multiplied with the purchasing power parity (PPP) dollar value for 2017 (World Bank, 2020). Cost benefit analyses were based on crop specific grain price averages and country specific input prices in addition overall averages.

## 3 Results

### 3.1 Nutrient main effects

The overall mean effect of P was found to be highly significant (p < 1e^−6^) with an average yield gain of 251 kg ha^−1^ at an average rate of 15 kg P ha^−1^. The average effects of K and SMN were estimated at 15 and 49 kg ha^−1^ respectively, and were not significant (p > 0.1). The estimated means for yield with and without inputs in the four crops are given in Table 1. Most of the extra yield achieved with inputs was due to the addition of phosphorus. In terms of overall grain yield, it seems that cowpea lagged the other three crops, while climbing bean produced the largest yields. The main effect of crop or the interaction with crop and any of the nutrients was not significant however, possibly due to the large degree of confounding between crop and country, which is an intrinsic feature of the data.

**Table 1:** Estimated means for yields (kg ha^−1^) of grain legumes in control plots and with application of P, K and SMN (i.e. secondary and micro-nutrients).

**Crop**	**no inputs**	**P only**	**P + K**	**P + K+SMN**	**Max. SE**
Soybean	1446	1746	1748	1800	240
Groundnut	1259	1477	1512	1531	255
Cowpea	945	1184	1068	1161	292
Climbing bean	1657	1905	2045	2076	315

**Table 2:** Effect of different subsets of variables on OOB R^2^.

**Type**	**control**	**P**	**K**	**SMN**
All explanatory	0.39	0.06	0.33	0.14
Coordinates only	0.23	0.05	0.20	0.09
Explanatory survey	0.35	0.11	0.30	0.17
Explanatory remote sensing	0.25	0.04	0.23	0.13
All predictive	0.31	0.04	0.28	0.13
Predictive survey	0.23	0.04	0.18	0.08
Predictive remote sensing	0.26	0.04	0.24	0.14

### 3.2 Yield and nutrient response variation

Considerable variability was observed for yield and nutrient responses. Summing over all hierarchical variance components (experiment, district, district/year and farm), the standard deviations associated with control yield, P, K and SMN response were 683, 190, 115 and 125 kg ha^−1^, respectively.

**Fig. 2:**
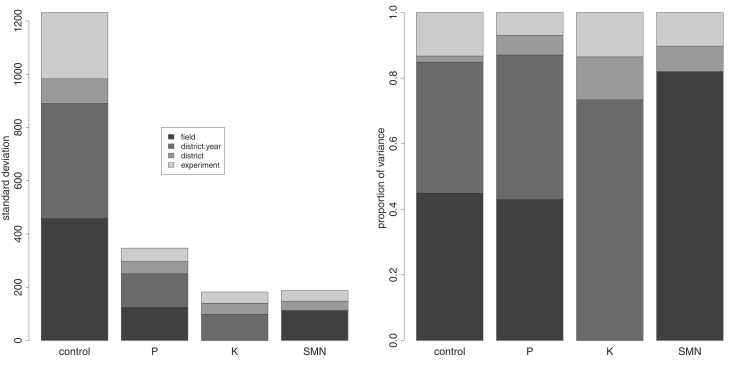
Left panel: breakdown of random variation at different strata (expressed as standard deviation) for control yield, P, K and SMN. Right panel: variance components expressed as proportions of total variance (excluding residual variance).

Breakdown into hierarchical components (Fig. 2) shows that in general, only a small amount of variation is associated with the predictable district level, with most variation found at the district/year, farm and to a lesser extent the experiment level. Only 2%, 6%, 13% and 8% of total variance, excluding residual variation, was found at the district level for control yield, P, K and SMN response. In terms of nutrient responses, 80% of districts are expected to have yield gains falling within 193–309, -37–67 and 5–93 kg ha^−1^ for P, K and SMN respectively, suggesting that there is little to be gained from district-specific recommendations.

Regarding the unpredictable components of variation, the first thing to notice is that despite substantial variation in control yield between experiments, there is very little variation at this level for any of the nutrient responses. This suggests that the effects of P, K and SMN are relatively stable across experiments performed with different crops over different countries and years, at least when similar procedures are followed as was the case here. Second, whereas the district/year and farm level represent similar amounts of variation in the case of control yield and P response, there is no variance associated with the farm level for K response and only a negligible amount of variance at the district/year level for SMN response. While theoretically, this could be due to large-scale and seasonal differences affecting K response and variation in secondary and micronutrients reflecting local deficiencies, it could also be an artefact of the overall small degree of variation in response for these two types of nutrients.

**Fig. 3:**
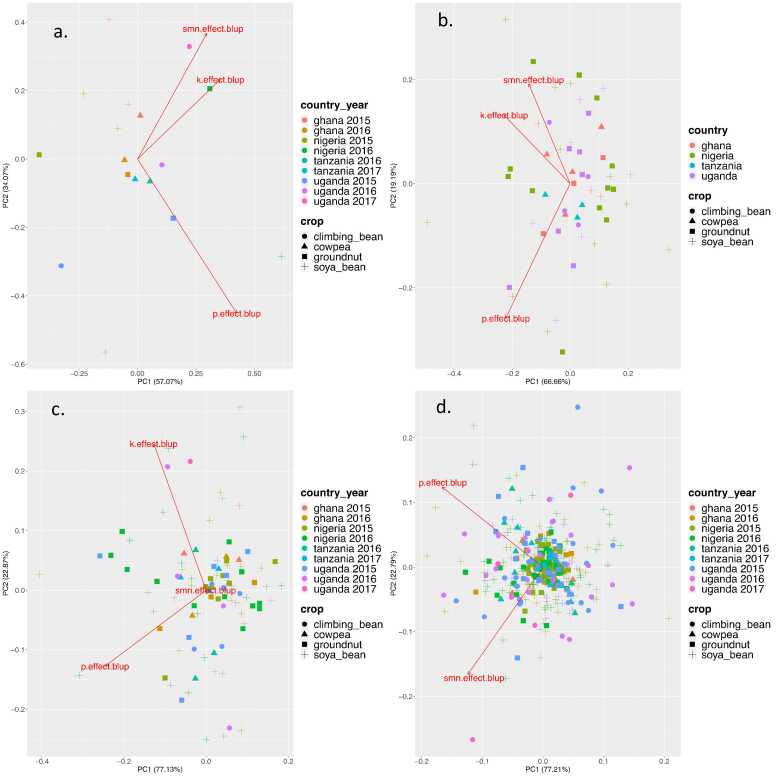
Biplots showing the principal components (PCs) and loadings corresponding to the first PCs calculated for the matrix of BLUPs for the response to P, K, and SNM. Panel a: experiment level, b: district level, c: district.year level and d: field level.

Dissecting the observed variability by principal component analysis of the random effects (BLUPs) at different hierarchical levels revealed that at the level of experiment, the 2016 Nigeria groundnut trial projects strongly on the K and SMN vectors (Fig. 3). This is confirmed by significant main effects for K and SMN in these trials, with 88 and 118 kg ha^−1^ of additional yield respectively (Ca, Mg, Zn, S, B). At the other extreme, the 2015 Ugandan climbing bean trials saw negative responses across all districts, leading to a significant negative main effect of the combination of Mg, Zn, Mo. The strong projection of the 2016 Nigeria soybean study on the P response vector corresponds to an estimated response of 456 kg ha^−1^.

At the district level, a few districts show a strong positive projection on the K and SMN vectors, Kwayakusar (Nigeria, soybean) and Kajuru (Nigeria, soybean and groundnut), in particular. For the latter district this corresponds to random effects of more than 20 kg ha^−1^ above the means of 15 and 49 kg ha^−1^ for K and SMN, respectively. Random effects for district level P responses range from − 17 kg ha^−1^ (Apac, Uganda, soybean) to 37 kg ha^−1^ (Kajuru, Nigeria soybean), with respect to the overall mean of 251 kg ha^−1^. For all three types of nutrients, the absolute deviations from the mean are minor, reflecting the limited level of variation at this level of hierarchy. In contrast, variation in P and K response at the district/year level is much more pronounced, with random effects for P and K ranging from -117–246 and -107–140 kg ha^−1^ respectively. This translates into several significant interactions between P and district, namely in Nigerian groundnut trials in 2015 and 2016 and Ugandan soybean trials in 2017, and between K and district in Ugandan bean trials in 2016 and Ugandan soybean trials in 2017. Similar variation in P response is found at the farm level, with random effects ranging from -237–2011 kg ha^−1^ above the mean. It is also at this level that the largest variation for response to SMN is observed with random effects from -110–81 kg ha^−1^ with respect to the mean.

### 3.3 Economic benefits

**Fig. 4:**
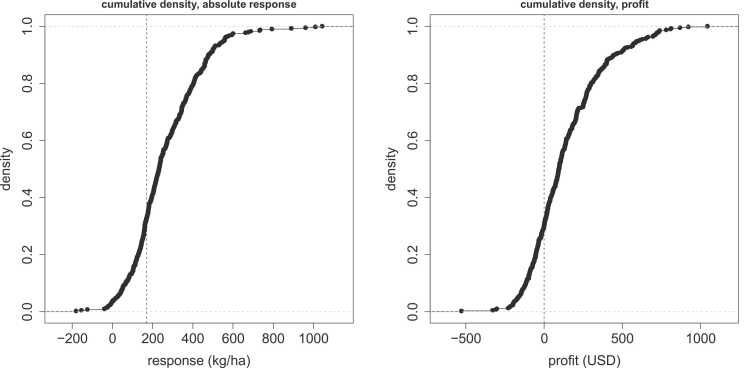
Cumulative densities of absolute response (left) and profitability (right) of P application. Vertical lines mark economical minimum rate (average prices across countries and legumes) and 0 profit (country- and legume-specific prices) respectively.

The considerable response variation for P has direct economic relevance to farmers: although the application of P-fertiliser increased legume yields consistently, the observed variation at the farm level will translate in unpredictable economic benefits. Fig. 4 summarises the total variation in P response at the farm level and its relation to profitability. The left panel shows the variation in absolute agronomic response with respect to the minimum response required to be profitable given a mean application of 15 kg ha^−1^ of P with average costs and grain prices across countries and legumes. Out of all fields, 96% showed an increase in yield in response to the application of P and 67% of fields had responses above the average economic minimum, meaning that a third of fields would fail to benefit from P application at the tested rates. Similarly, the actual distribution of estimated profits, using country and legume specific prices, is shown in the right panel. Average profit from P application was 137 USD ha^−1^, and on only 30% of fields application was unprofitable, with 11% posting losses greater than 100 USD ha^−1^ against 48% having gains in excess of 100 USD ha^−1^. The lack of significant yield responses to K and SMN means that their application is unlikely to be profitable, which is why we did not include them here.

### 3.4 Patterns and predictability in yield and effect variation

Random forest was used to model farm-level variation in control yields and nutrient responses as a function of explanatory and predictive soil, climatic and agronomically relevant survey variables (Table S2). We were interested in the capacity of the total set of variables to explain and predict patterns of variation. Apart from the general question of how much variation could be either predicted or explained, the contribution of different types of variables to predictive and explanatory ability was also of interest, particularly the contribution of the survey variables, which are costly to collect compared to remote sensing data.

**Fig. 5:**
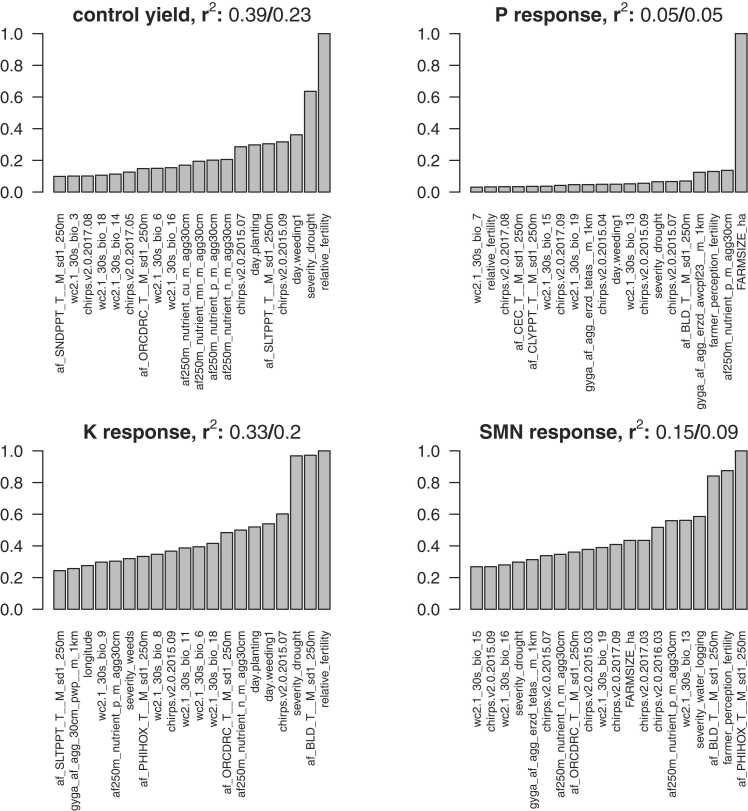
Histograms showing the distribution of relative variable importance (top 20 remote sensing and survey variables, Table S2) of random forest predictions of control yield and P, K and SMN response, using the full set of variables. The corresponding out of bag R squared values for the full model / model with coordinates only are shown in the plot titles.

Only a small proportion of variation was accounted for by the total set of variables (Fig. 5, Table 1) but overall, model accuracy was slightly higher for explanatory than for predictive models, indicating that season-specific information holds explanatory value. For both explanatory and predictive variables, model fits were particularly poor for response to P and SMN (with values of out-of-bag R squared below 0.15) and were better for control yield and response to K (R-squared above 0.33). It is worth mentioning that models with all variables included were only moderately better compared with a model with geographic coordinates only. Particularly in the case of response to P and SMN, adding variables other than latitude and longitude did not improve the model substantially. Although this suggests that individual variable importance should be interpreted with caution, the fact that reported soil fertility and drought severity were found among the three most important explanatory variables for both control yield and K response (Fig. 5) could point to water and nutrient availability as potential shared constraints. Survey and remote sensing variables were found to be complementary for control yield and K response, with models containing both having higher accuracies than those with only a single type of variable. This was the case for both explanatory and predictive models but, in case of the former, survey variables seemed to contribute more information while in the latter models using remote sensing variables were more accurate than those containing only survey variables. In all cases, however, their accuracy was only marginally better compared to those with coordinates only.

**Fig. 6:**
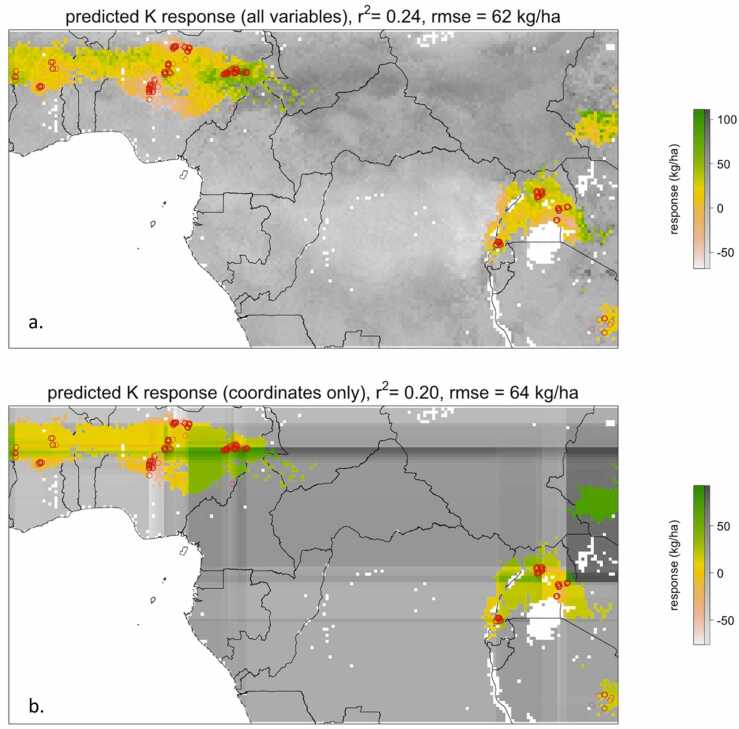
Two maps of the predicted absolute response to K (in kg/ha) showing the different spatial patterns produced by a Random Forest model using all remote sensing variables (a., 24% of variation explained, rmse of 62 kg/ha), and geographic coordinates only (b., 20% of variation explained, rmse of 64 kg/ha). Trial sites are shown in red. Predictions for representative areas (i.e., those with a predicted probability of representing a potential trial site of >0.53) are highlighted in full colour. The grayscale background shows predictions for non-representative areas and is included for appreciation of spatial structure of predictions only.

In theory, predictive models such as those evaluated above could contribute to the development of context-specific nutrient recommendations, in which nutrients are targeted to areas where they are predicted to be most effective. While attractive, such an approach has important caveats and requires spatial predictions to be accurate, reliable and of sufficient amplitude. We use the predictive model for K response, which had relatively good accuracy, as an opportunity to evaluate the potential and limitations for site-specific predictions. Fig. 6a shows what a map of predicted K response looks like, based on the model with all predictive geospatial variables, within the areas for which the trials were considered environmentally representative. The map shows quite distinct areas of stronger and weaker response, but the magnitude and amplitude of yield response variation is limited, with the upper 5% predicted K responses being 63 kg ha^−1^ compared to a median value of 22 kg ha^−1^ respectively. In addition, the reliability of the spatial predictions seems questionable. While the predictive model with coordinates alone explains almost as much variation as the full model, the spatial patterns of predictions differ substantially from those predicted by the full set of variables (Fig. 6b). This demonstrates that prediction accuracy cannot be credited to the environmental variables included in the model and suggests that spatial predictions probably vary depending on the available data.

## 4 Discussion

### 4.1 General response and profitability of P, K and SMN

Consistent and profitable nutrient responses are important if general recommendations are to be made. Among the three types of nutrients tested, only P was found to have a substantial and significant main effect, with a mean response of 251 kg ha^−1^. Neither K nor secondary and micronutrients were found to have substantial positive effects on average. At the same time, considerable variation in response was observed for all three types of nutrients, which reflects earlier findings in literature. For P, most published estimates of response in soybean, common bean, cowpea and groundnut tend to be between 150 and 500 kg ha^−1^ (Chekanai et al., 2018, Giller et al., 1998, Kaizzi et al., 2018, Kaizzi et al., 2012, Maman et al., 2017, Ronner et al., 2016, Serme et al., 2018, Tarfa et al., 2017, Ulzen et al., 2018, Zingore et al., 2008) and can be considered consistent with the 250 kg ha^−1^ found in our study, although responses below 100 kg ha^−1^ (Ikeogu and Nwofia, 2013, Mabapa et al., 2010, Serme et al., 2018, Smithson et al., 1993) and above 500 kg ha^−1^ (Kaizzi et al., 2018, Kamara et al., 2007, Moses et al., 2018, Tarfa et al., 2017) have also been reported.

At the tested application rate of 15 kg ha^−1^ on average, and considering only input costs, P application was profitable in terms of immediate response in almost 70% of cases, at a yield response of about 200 kg ha^−1^ (Fig. 4), an outcome which in the absence of systematic spatial variation can be considered representative for the study area. While such cost-benefit analyses need to be treated with caution, the present result suggests that the application of P could be generally beneficial but also highlights that economic risks may still be an issue to farmers, although losses in excess of 100 USD/ha were estimated to be rare. Most of the earlier published studies applied larger rates of 30 or 40 kg ha^−1^ P in soybean, and 15–20 kg ha^−1^ in groundnut, common bean and cowpea. No effect of larger application rates emerges from literature (for instance, Tarfa et al. (2017) reported an average response of 590 kg ha^−1^ to 15 kg P ha^−1^ in soybean, while Kaizzi et al. (2012) found a response of 180 kg ha^−1^ to 37.5 kg P ha^−1^. Moreover, it is likely that P addition beyond 20 kg ha^−1^ will be less profitable than what we report here, given that nutrient use efficiencies tend to decrease at higher rates.

For K, the insignificant average response reported here is consistent with other reports of responses below 100 kg ha^−1^, or even negative responses, in soybean, cowpea and groundnut (Kihara et al., 2017, Maman et al., 2017, Moses et al., 2018, Serme et al., 2018, Tarfa et al., 2017). Negative responses seem to occur in cases where K is not limiting, caused by a damage to plant roots (Kihara et al., 2017). Still, recent pot experiments in soybean (Baijukya et al., 2021) identified K as being potentially limiting on a number of soils collected in East and West Africa and there are several field studies reporting grain yield responses between 100 and 300 kg ha^−1^ for groundnut, common bean and cowpea (Moses et al., 2018, Serme et al., 2018, Smithson et al., 1993, Tarfa et al., 2017) and even an exceptional 700 kg ha^−1^ in common bean (Kaizzi, 2018). Such studies may allude to either localised deficiency of K (cf. Smithson et al., 1993), though in our study we did not find consistent responses at the location level (see also 4.2), or to weather dependent effects (Martineau et al., 2017), creating exceptional circumstances.

Similarly, other nutrients affected yields in particular cases, but not consistently, in line with the limited response to S and micronutrients (Kihara et al., 2017) and to Mg-S-Zn-B (Serme et al., 2018, Wortmann et al., 2019). Where responses to K and SMN were larger than average, the magnitude was still small compared with the response to P.

### 4.2 Variability and predictability of nutrient response variation

Variability of control yields and nutrient responses represent production risk that may hamper investment in nutrient inputs, unless this variability can be predicted. Our results suggest that only a relatively small part of variation can be explained or predicted by geographic, environmental or survey variables that are commonly collected. This confirms earlier results showing the lack of explainable patterns in agronomic outcomes (Ronner et al., 2016). In terms of geography, variation in nutrient response in our study was predominantly associated with the non-predictable levels of district/year and field, rather than at the predictable district level. The relatively limited variability in nutrient response at the level of experiment suggests that strong differences between the published studies are not explained by random trial-to-trial variation but rather reflect systematic differences in implementation (e.g., addition of manure or other nutrients, differences between varieties, plant densities, etc.), or could be due to studies reporting on a limited number of locations.

Similarly, we found that relatively little variation in nutrient response could be explained by our total set of covariables. Only K response could be modelled reasonably well, although the maximum proportion of variation explained was still limited and considerably below the 40% observed for control yield. Still, the result is indicative of relatively strong influence of geography and/or environment on observed response variation. Interpretation of such patterns is not straightforward, however. The relatively large variance component for district/year and the strong contribution of precipitation and drought related variables to variation suggest that seasonal effects play an important role. Since seasonal effects are unpredictable, they are of little relevance for local nutrient adjustments. In this regard the amount of variation that can be predicted from time independent covariates such as climatic and soil parameters are more important. We found K response to be relatively well predicted by those variables, which in theory could help in generating regional recommendations on K use. Our exploration of the patterns and stability of spatial predictions highlights the limitations of such an approach, however. The fact that maps of spatial predictions based on a full set of variables and on geographic coordinates alone differ substantially, while explaining similar amounts of variation, suggested that large-scale spatial patterns predicted from on-farm trials are not necessarily reliable and should not be taken at face value. In addition, regardless of the reliability of spatial patterns, only 5% of sites had predicted yield response of more than 63 kg ha^−1^, an outcome that is unlikely to justify any adjustment from general recommendations. Combined with the limited accuracy, i.e. 20% of variance explained, it seems that effective tailored recommendations (Abera et al., 2022, Chivenge et al., 2022, Ebanyat et al., 2010, Vanlauwe et al., 2015, Zingore et al., 2008) are not warranted based on our data and may be difficult to achieve in practice (van Heerwaarden, 2022).

In terms of the contribution of different types of variables to the explanation and prediction of yield and response variation, several things are worth noting. First, as concluded above, our selected set of remote sensing variables seemed to add little information to that represented by geographic coordinates alone. While adding such variables produced distinct and perhaps more realistic-looking spatial prediction patterns, no superiority for such predictions can be claimed if they do not explain substantial amounts of additional variation. This was the case for control yield as well as for the three types of nutrient responses and may indicate that available remote sensing data lacks resolution and accuracy to be of value or, alternatively, that we have failed to identify more relevant covariates that are available through remote sensing. Second, none of the covariates, be it remote sensing or survey variables, had much predictive power over geographic coordinates alone. This demonstrates the difficulty in predicting yields and crop responses based on prior information. Finally, in terms of explanatory power, survey variables contributed most of the relevant information. This suggests that information obtained directly from farmers helps to explain observed agronomic outcomes better than remote sensing information alone, although researchers should decide if the added benefit in explanatory power outweighs investments in on-site data collection.

## 5 Conclusion

Our results confirm the general cost-effectiveness of P application in legumes, although with some economic risk. We did not find consistent evidence of a general positive response to either K or secondary and micronutrients but did observe substantial spatio-temporal response variation for these inputs. Limited predictability and low magnitude of predicted variation mean that effective tailored recommendations are not warranted based on our data. Therefore, it appears that nutrient use efficiency could be increased more effectively by investing in research on improved timing, placement and composition of fertilizer (van Heerwaarden, 2022). We demonstrate that despite limited ability to statistically model yield and response, the inclusion of selected survey questions improved our ability to explain the observed variation, suggesting that they may add valuable agronomic information that is not captured by geographic and remote sensing data. Future research should determine wether more informative on-farm variables and more cost-effective ways of obtaining these at scale can help understand and manage variability and risk in smallholder agriculture.

## Declaration of Competing Interest

The authors declare that they have no known competing financial interests or personal relationships that could have appeared to influence the work reported in this paper.

## Data Availability

Data will be made available on request.

## Appendix A Supplementary material

Supplementary materialTable S 1 Full overview of nutrient treatments and rates used in the different experiments.

.

Supplementary materialTable S 2 List of variables used in the random forest analysis
